# Control Strategies and Performance Assessment of Upper-Limb TMR Prostheses: A Review

**DOI:** 10.3390/s21061953

**Published:** 2021-03-10

**Authors:** Federico Mereu, Francesca Leone, Cosimo Gentile, Francesca Cordella, Emanuele Gruppioni, Loredana Zollo

**Affiliations:** 1 Unit of Advanced Robotics and Human-Centred Technologies, Università Campus Bio-Medico di Roma, 00128 Rome, Italy; f.mereu@unicampus.it (F.M.); f.leone@unicampus.it (F.L.); c.gentile@unicampus.it (C.G.); f.cordella@unicampus.it (F.C.); 2 INAIL Prosthetic Center, 40054 Vigorso di Budrio, Italy; e.gruppioni@inail.it

**Keywords:** Targeted Muscle Reinnervation (TMR), upper limb amputee, prosthesis, prosthetic control, multi-DoF control, pattern recognition

## Abstract

The evolution of technological and surgical techniques has made it possible to obtain an even more intuitive control of multiple joints using advanced prosthetic systems. Targeted Muscle Reinnervation (TMR) is considered to be an innovative and relevant surgical technique for improving the prosthetic control for people with different amputation levels of the limb. Indeed, TMR surgery makes it possible to obtain reinnervated areas that act as biological amplifiers of the motor control. On the technological side, a great deal of research has been conducted in order to evaluate various types of myoelectric prosthetic control strategies, whether direct control or pattern recognition-based control. In the literature, different control performance metrics, which have been evaluated on TMR subjects, have been introduced, but no accepted reference standard defines the better strategy for evaluating the prosthetic control. Indeed, the presence of several evaluation tests that are based on different metrics makes it difficult the definition of standard guidelines for comprehending the potentiality of the proposed control systems. Additionally, there is a lack of evidence about the comparison of different evaluation approaches or the presence of guidelines on the most suitable test to proceed for a TMR patients case study. Thus, this review aims at identifying these limitations by examining the several studies in the literature on TMR subjects, with different amputation levels, and proposing a standard method for evaluating the control performance metrics.

## 1. Introduction

The amputation of the upper limb causes a huge decrease in dexterity, with a significant reduction in patients’ quality of life. People who have had an upper-limb amputation need a prosthesis that replaces the lost arm functionality. It is very difficult to find epidemiology data on amputations of the upper limb. Over the world, the population of amputees was estimated as 10 million, 30% of whom are upper limb amputees [1]. Focusing on European countries, in Italy, there were 2720 upper-limb amputations in 2018, being equal to 18% of total amputations [2]; in 2003, in the UK, there were 5767 new amputations, 5% were upper limb amputees [3]. Between 2004 and 2013, only in the adult hand emergency clinic of the Nancy University Hospital (France), 2247 patients suffered an upper limb amputation (partial and pediatric amputation excluded) that was traumatic in 76.32% of cases [4]. In the USA, approximately 340,000 people have also suffered the loss of a limb and every year there are 10,000 new upper limb amputations, as reported by the National Center for Health Statistics [5]. The relevance of the upper limb loss has pushed international research to seek new prosthetic solutions [6,7].

Prosthesis technology ranges from passive or cosmetic typologies on one end to active or functional types on the other. Cosmetic prostheses are used to only restore the aesthetic aspect [8], while active ones are used to restore, as far as possible, the functionality of the lost arm. Active prostheses can be further classified into body powered, that exploit cables to control the device with the more proximal joints, and externally powered (electric or pneumatic) [9], which allow for the movement of the motors of the joints making up the prosthesis [10]. Externally powered prostheses require a control system in order to associate an input signal (generated by a sensor and/or a button) to an output action. One of the most used control systems is the myoelectric one, which exploits the electromyographic (EMG) signals of a specific muscle to provide discrete movement and of an antagonist muscle group to make complementary movements. EMG signals have been used to control prostheses since 1948 [11] and, over the years, various control strategies have been identified: among these, the control strategies that directly associate a movement of the prosthetic limb to an EMG input signal are usually referred to as Direct Myoelectric Control or simply Direct Control (DC). The conventional techniques, to control from one Degree of Freedom (DoF) to multiple DoFs [12], are [13]: the on/off strategy, which is typically used to control one DoF and allowing the performance of two opposite movements based on the exceeding of a preset threshold by the EMG amplitude of two residual antagonist muscles; the proportional control strategy that considers, instead, the voltage applied to the motor proportional to the contraction level/intensity of EMG signals.

**Figure 1:**
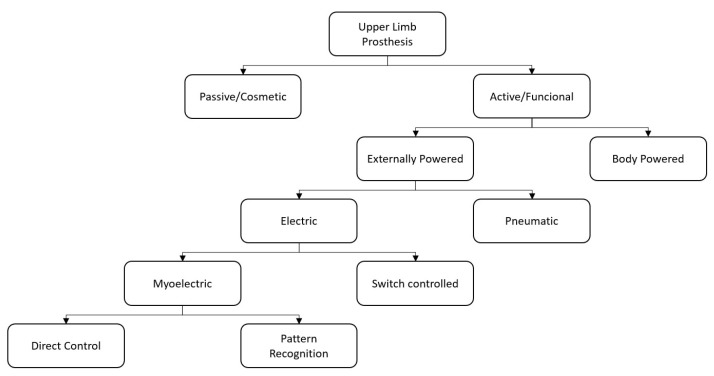
A Block diagram describing the types of Upper Limb Prostheses and control approaches.

Such control strategies are generally associated with a method for selecting the joint to be controlled. The co-contraction method is the first one, which allows the patient to change from one joint to another by simultaneously contracting the muscles used to control the joint; however, the principal limit of this technique is that it is possible to only control one joint at a time. The second is the simultaneous method that is used to control multi-DoF prostheses, handling more than one joint at the same time. However, in this case, the number of controllable DoFs depends on the number of independent EMG control sites [6] Figure 1.

The Pattern Recognition (PR) based myoelectric control has been proposed to reach a more intuitive and adaptable control of the prosthesis in order to overcome these limits and eliminate the need for mode switching; these PR strategies do not require independent muscle sites, but they consider muscular activation patterns of different muscle sites to classify several motion classes. In spite of the progress made in this field based on EMG-PR [14], the main reasons for the abandonment of the prosthetic device by many users can be imputed to comfort and function [15]. Regarding the function, technological factors, as the type of prosthetic control and the presence or absence of sensory feedback [16], have a key role in avoiding the decision of prosthesis abandonment. An indispensable requirement is that the control system must be simple, direct, and user-friendly [17].

To reach these requirements, a major advancement in the field of upper-limb prosthetics has been reached with the Targeted Muscle Reinnervation (TMR) surgery, which was developed by Prof. Todd Kuiken and his team at the Rehabilitation Institute of Chicago [18]. The idea behind TMR was that reinnervating the residual nerves of the amputated limb to new target muscles may allow users to more intuitively control the prosthesis and simply perform Activities of Daily Living (ADL). This is because the patient’s intention, manifested with the phantom limb, can be sent, as neural information, to the reinnervated muscle, which amplifies the EMG signal that is used to control the prosthetic device.

Several control strategies were proposed in the literature for making myoelectric prostheses control easy, reliable, efficient, and, therefore, for lowering the users’ cognitive burden for TMR patients. However, the optimal control system that allows users to control a multiple DoF prosthesis with dexterity, and by using intuitive interfaces between the user and the device, has not yet been developed [19].

## 2. Targeted Muscle Reinnervation

**Figure 2:**
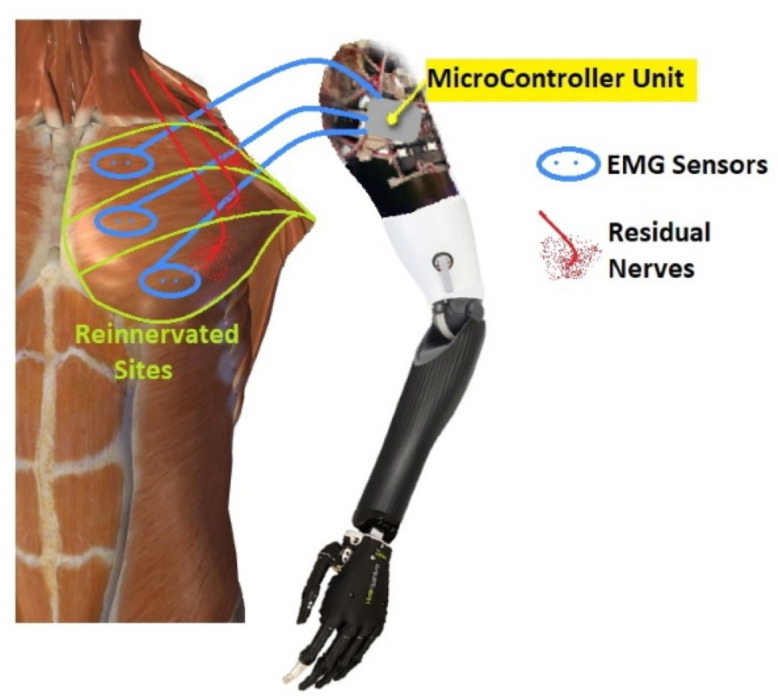
An example of a prosthesis control system after Targeted Muscle Reinnervation (TMR) surgery: the electromyographic (EMG) sensors collected from the reinnervated sites the EMG signals and communicated to the MicroController Unit (MCU) the user’s intention to translate into arm and hand movements.

After an upper-extremity amputation, the employment of TMR allows for improving the functionality of myoelectric prostheses: the reinnervation of residual muscles creates additional myoelectric control sites available for obtaining the multi-DoF prosthetic control, without the need of switching between modalities available on the device [20]. In 1995, Kuiken examined muscle recovery and related changes in the motor unit population of “hyper-reinnervated” rats [18]. Only in 2004, the first TMR surgery was performed on one human subject with bilateral shoulder disarticulation amputation [21] Figure 2.

**Figure 3:**
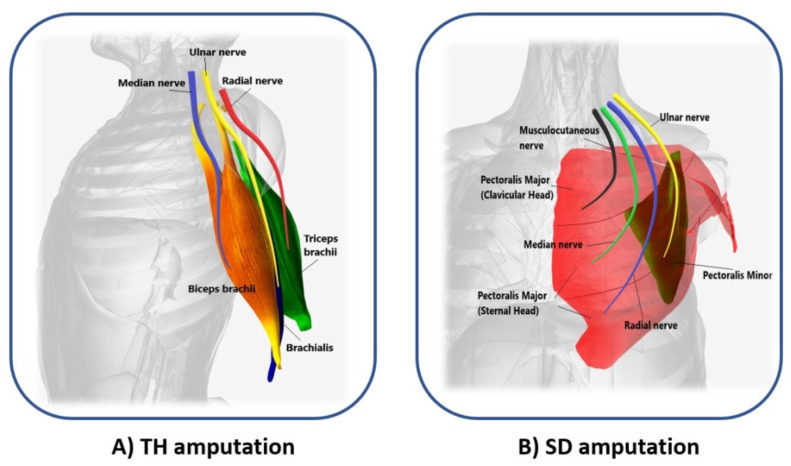
Scheme of the reinnervated sites for different levels of amputation. (**A**) Median (blue), ulnar (yellow), and radial (red) nerves transfer on biceps brachii (orange), brachialis (violet), and triceps brachii (green) muscles of transhumeral (TH) amputees; (**B**) Musculocutaneous (black), median (light green), radial (blue), and ulnar (yellow) nerves transfer on pectoralis major (clavicular and sternal head, in red), and pectoralis minor muscles (dark green) of shoulder disarticulation (SD) amputees.

In 2006, Kuiken introduced the following requirements to make TMR surgery successful: (i) separate regions of muscles and skin must be reinnervated by multiple donor nerves; (ii) EMG signals must be acquired from each target area; and, (iii) the prosthesis must be able to receive numerous EMG input signals and control several motors [22]. TMR can be performed for three different levels of amputation: shoulder disarticulation, transhumeral, and transradial amputation. The innervation strategies depend on the type of amputation [23]. For the shoulder disarticulated patients Figure 3B, pectoralis muscles are usually denervated and then reinnervated with residual arm peripheral nerves [22]. Afterward, back muscles (if possible) are also reinnervated to have more active sites. For the transhumeral amputees Figure 3A, the median nerve is transferred to the short head of the biceps motor branch to restore the function of hand closing or pronation; the ulnar nerve is transferred to a residual brachialis motor branch to have additional control sites for hand closing; finally, the radial nerve is reinnervated to the lateral head of the triceps motor branch in order to control hand opening or supination [24]. For transradial amputees, the control of multifunctional prosthetic hands can be reached by using additional Targeted Muscle Reinnervation signals for improving the function of intrinsic finger and thumb muscles: the distal median nerve is transferred to the flexor digitorum superficialis, while the ulnar nerve is reinnervated to the flexor carpi ulnaris [25]. When the muscles usually chosen cannot be reinnervated, as in [26], three bundles of the anterior tight muscle are used to obtain three active sites for the prosthetic control. The TMR is also an emerging technique for the treatment and reduction of the phantom limb pain (PLP) and neuroma pain [27], for the osseointegrated prostheses [28], and for the targeted sensory Reinnervation [29] of bidirectional neuroprosthetic devices. Finally, another important outcome is the use of TMR in the oncologic population, due to the potential to reduce pain without the use of opioids [30].

## 3. Aim of the Study

This paper proposes an in-depth study of the literature on control strategies for prostheses that were developed for amputee subjects who underwent TMR procedure. The scope is to consolidate the current knowledge in this field and delineate the limits of these strategies that, up to now, do not yet allow for natural and simultaneous control of the prosthetic arm DoFs.

Nowadays, according to the literature search, the related review papers on TMR contributed to defining the advantages of using this surgery technique from a medical perspective [23,30] without analyzing the most suitable control strategies and performance evaluation tests that allow exploiting the additional targeted muscle reinnervation sites to improve the multi-DoF prosthesis control.

This work has the twofold purpose of (i) identifying the main issues and advantages of the control strategies that were proposed in the literature in order to address the future research towards the development of prostheses that are functional and able to mimic the lost upper limb behavior, replicating the performance of the human arm, for amputee subjects who have undergone the TMR procedure; and, (ii) suggesting a unified protocol test for the validation of these control strategies and, in the case of PR, in both offline and online mode. The expected added value provided by this work is to complete the current knowledge on the control strategies with more recent papers, by critically evaluating and comparing (when possible) the available results and pointing out inconsistencies and neglected aspects.

The paper is organized, as follows. Section 3 describes the methods that were used to select the reviewed articles. Section 4 introduces the benefits of TMR. Section 5 describes the control strategies (both DC and PR) that were used in the analyzed papers for TMR patients. In Section 6, the performance evaluation methods are reported. Section 7 underlines the principal limits of the current control strategies and suggests a unified protocol for the control performance evaluation. Finally, conclusions are drawn in Section 8.

## 4. Materials and Methods

A wide search was conducted through the following databases: PubMed and Google Scholar. The search terms included the following keywords and their combinations: Targeted Muscle Reinnervation (TMR), upper limb, prosthesis, amputation level, prosthetic control, real-time and offline performance, multi-DoF control, and pattern recognition. Only studies that were published between 2004 and February 2020 were selected.

All of the found articles were in English and available in full text on peer-reviewed journals or in conference proceedings. Some additional papers extracted from the references of the examined articles have been included.

The authors reviewed all articles fulfilling the following inclusion criteria:

1. Be a study on upper limb prosthesis users that underwent TMR surgery.
2. Concern control techniques in upper limb prosthesis.
3. Involve both direct and pattern recognition control strategies.
4. Use methods for evaluating the performance of the prosthetic control.
5. Be a full-length publication in a peer-reviewed journal or in conference proceedings.

**Figure 4:**
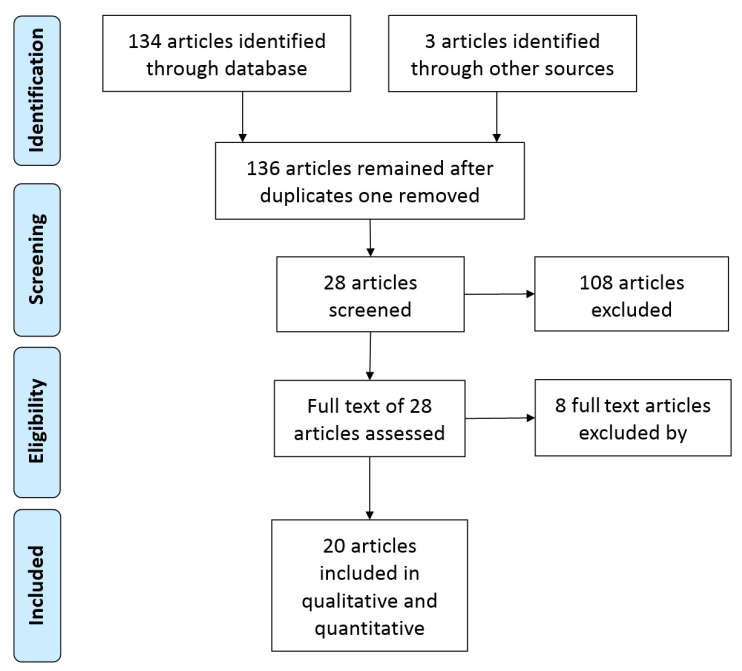
Flow diagram of the search and inclusion process.

The search strategy was based on the PRISMA (Preferred Reporting Items for Systematics reviews and Meta-Analyses) statement (2009), as shown in Figure 4.

A total of 136 papers was analyzed by using the previously mentioned search method. After considering the titles and abstracts, 108 articles were excluded from the initial 136, because they did not meet the inclusion criteria. The remaining 28 articles have been carefully analyzed. Eight of them were further excluded because the reported data were not significant or were repetitive for the purpose of this work. This review discusses the remaining 20 articles.

The selected studies have been classified into two main groups according to the used control strategy (DC or PR); one more group was devoted to papers on comparison among strategies:

1. Direct Control strategy—six papers: Kuiken et al. (2004) [21], Kuiken et al. (2005) [31], Kuiken et al. (2007) [24], Miller et al. (2008) [32], O’Shaughnessy et al. (2008) [33], and Miller et al. (2008-b) [34]
2. Pattern Recognition strategy—10 papers: Mastinu et al. (2018) [28], Kuiken et al. (2009) [35], Smith et al. (2013) [36], Huang et al. (2008) [37], Zhou et al. (2007) [38], Batzianoulis et al. (2019) [39], Batzianoulis et al. (2018) [40], Xu et al. (2018) [41], Hargrove et al. (2018) [42], and Tkach et al. (2014) [43].
3. Comparison of different types of control—four papers: Hargrove et al. (2013) [44], Wurth and Hargrove (2014) [45], Hargrove et al. (2017) [46], and Young et al. (2014) [47]

The following information has been extracted from the studies and reported in Table 1:

- the number of the enrolled patients;
- the amputation level: bilateral shoulder disarticulation (BSD), shoulder disarticulation (SD), and transhumeral (TH);
- the number of reinnervated sites/control sites after the TMR surgery;
- the use of prostheses/virtual reality (VR);
- the number of controllable DoF/motion classes; and,
- the adopted performance evaluation methods.

**Table 1:** Summary of the reported analysis.

Study	No. of Patients	Amp. Level	No. of Reinnervated Sites/Control Sites	Prostheses/ Virtual Reality	DoF/ Motion Classes	Performance Evaluation Methods
Kuiken et al. [21]	1	BSD	4 reinnervated sites/ 3 muscle control sites	Prosthesis—DC	2	BBT, CRT
Kuiken et al. [31]	2	BSD, TH	4 reinnervated sites/ 3 muscle control sites (BSD) 2 muscle sites (TH)	Prosthesis—DC	2	BBT, CRT, WMFT, AMPS
Kuiken et al. [24]	1	TH	4 muscle sites and 2 sensory sites	Prosthesis—DC	2	BBT, AMPS, light touch, graded pressure, texture, edge detection, and thermal feedback
Miller et al. [32]	1	BSD	4 reinnervated sites	Prosthesis—DC	3	BBT, CRT, Cubbies, Cups
O’Shaughnessy et al. [33]	3	TH	2 reinnervated sites/ 4 control sites	Prosthesis—DC	2	BBT, CRT, AMPS
Miller et al. [34]	6	SD, TH	2 reinnervated sites (TH), 4 reinnervated sites (SD)	Prosthesis—DC	2	BBT, CRT, AMPS
Mastinu et al. [28]	2	TH	2 reinnervated sites	PR without prosthesis	4 discrete hand and elbow motions	accuracy offline, classification error rate of LDA with 4 time domain features (MAV, WL, ZC, SSC)
Kuiken et al. [35]	5	SD, TH	4 reinnervated sites, 4 control sites	PR without prosthesis—VR	10 discrete elbow, hand and wrist motions	accuracy offline, motion selection time, motion completion time, and motion completion rate of LDA with TD features [38]
Smith et al. [36]	5	SD, TH	3–4 reinnervated sites (SD1, SD2), 2 reinnervated sites (TH)	PR without prosthesis	9 discrete elbow, hand and wrist motions	classification error rate of LDA with TD features [48]
Haung et al. [37]	3	BSD, TH	4 reinnervated sites (BSD), 4–2 reinnervated sites (STH, LTH)	PR without prosthesis	15 discrete elbow, hand and wrist motions	offline accuracy of LDA classifier with TD features (MAV, ZC, SSC, WL)
Zhou et al. [38]	4	BSD, STH, LTH	4 reinnervated sites (BSD), 4–2 reinnervated sites (STH, LTH)	PR without prosthesis	16 discrete movements of the arm, hand, and finger/thumb	offline accuracy of LDA classifier with TD feature set, and a combination of AR-RMS
Batzianoulis et al. [40]	2	TR	TMR surgery for the neuroma pain, not for control sites	PR without prosthesis	5 grasp types (prismatic-2 fingers, precision disk, palm pinch, lateral, prismatic-4 fingers)	offline accuracy, standard errors of LDA, two SVMs, and ESN Network
Batzianoulis et al. [39]	2	TR	TMR surgery for the neuroma pain, not for control sites	PR without prosthesis	3 grasp types (precision disk, lateral, and palm pinch)	offline accuracy of LDA classifier with TD feature
Xu et al. [41]	1	TH	3 reinnervated sites/ 5 control sites	Prosthesis—PR	6 discrete elbow, wrist and hand motions	offline accuracy, ARAT, LDA classifier with TD features (MAV, WL, ZC, SSC)
Hargrove et al. [42]	9	TH	not described	Prosthesis and VR—PR	6 discrete elbow, wrist and hand motions	SHAP, JTHFT, CRT, BBT, ACMC, the classification error rate, completion time, failure rate of LDA classifier with TDAR
Tkach et al. [43]	4	SD, TH	4 reinnervated sites (TH), 2 reinnervated sites (SD)	PR without prosthesis—VR	8 discrete and combined elbow, wrist and hand motions	offline accuracy of the LDA classifier with AR feature set
Hargrove et al. [44]	4	SD, TH	4–5 reinnervated control sites	Prosthesis—DC and PR	2 DoFs (sequentially PR system)	BBT, BST, CRT, classification error rates
Wurth et al. [45]	1	TH	4 control sites	PR and DC without prosthesis—VR	2 DoFs (sequentially and simultaneously PR systems)	FTAT, throughput (bits/second), path efficiency (%), completion rate (%)
Hargrove et al. [46]	8	TH	4 control sites	Prosthesis—DC and PR	2 DoFs	ACMC, SHAP, BBT, CRT
Young et al. [47]	3	SD, TH	2 reinnervated sites/ 4 control sites	Prosthesis—DC and PR	2 DoFs (sequentially and simultaneously PR systems)	TAC test (completion time, completion rate, length error), offline classification error

Acronyms of Table 1: BSD: Bilateral Shoulder Disarticulation; SD: Shoulder Disarticulation; TH: Transhumeral; LTH-STH: Long (L)–Short (S)Transhumeral; TR: Transradial; DC: Direct Control; PR: Pattern Recognition; VR: Virtual Reality; BBT: Box and Block Test CRT: Clothespin Relocation Test; WMFT:Wolf Motor Functions Tests; AMPS: Assessment of Motor and Process Skills; LDA: Linear Discriminant Analysis; MAV: Mean Absolute Value; WL: Waveform Length; ZC: Zero Crossing; SSC: Slope Sign Changes; TD: Time Domain; AR-RMS: Auto Regressive-Root Mean Square; ESN: Echo State Network; SVM: Support Vector Machine; TD-AR: Time Domain and Auto Regressive; ARAT: Action Research Arm Test; BST: Block stacking test; FTAT: Fitts’ Target Acquisition Task; SHAP: Southampton Hand Assessment Procedure; JTHFT: Jebsen-Taylor test of Hand Function.

## 5. Control Strategies

### 5.1. Direct Control

**Figure 5:**
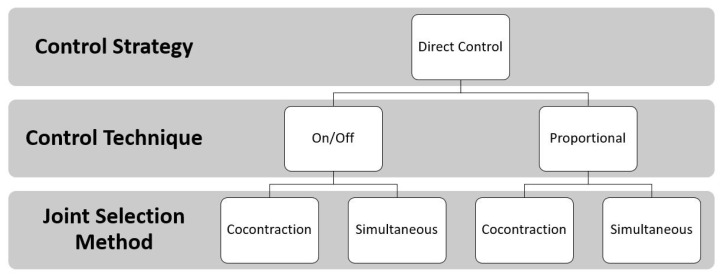
Direct Control approach: the EMG signals are the input to the controller unit. Two control techniques (the on/off and the proportional) defined the speed necessary to move the joint when the EMG signal is above a predefined threshold. The joint selection methods allow for the user to switch joints with muscle co-contraction or to select them simultaneously.

The control strategies where EMG signals are directly associated with a specific movement are named direct control strategies, as mentioned in Section 1. Among them, the most used are on/off and proportional techniques. Multiples control techniques can be combined with the joint selection method to control multi-DoF prostheses. Figure 5 shows the DC approach.

**Figure 6:**
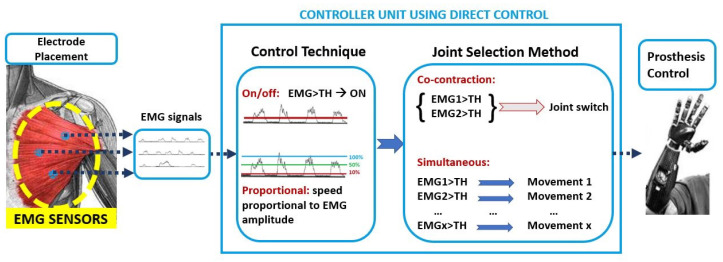
Schematic diagram of direct myoelectric control techniques and joint selection methods.

In detail, the control techniques indicate the relationship between the value of the input signal and the value of the output. In the on/off technique there are only two possible output signals: a predefined speed value (on) and zero (off). The input signal must exceed the preset threshold in order to generate the output value that is used for motor control. Instead, with the proportional technique, it is possible to create a proportional link between the motor speed (output) and the amplitude of the EMG signal (input). Of course, there is also a threshold below which the output signal is zero; in some cases, there is another threshold, above which the output signal is the maximum possible. Regardless of which control technique is used, when the EMG signals are fewer than the DoFs to be activated, the user can employ muscle co-contraction to choose the DoFs to be controlled with the same EMG signal, as mentioned in Section 1. The introduction of TMR surgery made possible to have more muscle sites to uniquely associate a sEMG signal with a movement and simultaneously control more than one DoF. This procedure is resumed in Figure 6.

**Figure 7:**
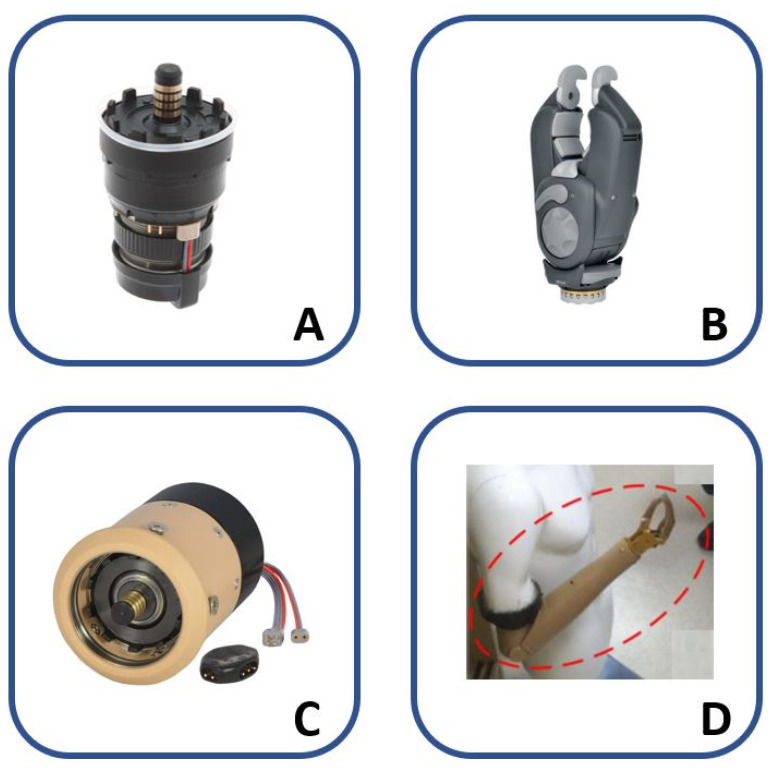
Prosthetic systems used in the analyzed papers on the TMR subjects: (**A**) Wrist rotator (Ottobock) used in [21,24,31,34]—Photo courtesy of Ottobock; (**B**) Greifer Terminal (Ottobock) used in [21,31,34,46]—Photo courtesy of Ottobock; (**C**) Wrist rotator (Motion Control Inc.) used in [42,46]—Photo courtesy of Motion Control, Inc.; (**D**) Danyang Prostheses Co. (Ltd, Danyang City, China) used in [41]— (http://creativecommons.org/licenses/by/4.0/, accessed on 5 March 2021), this image has been cut from [41].

The following six articles make a clear reference to the use of direct control following a TMR intervention. Particular attention was paid to information regarding the level of amputation, the number of sites reinnervated and adopted for the control, and the used prosthetic devices (when indicated are reported in Figure 7), in order to critically evaluate and compare the performance of each method, and to point out the most functional prosthesis control method.

In Kuiken et al. [21], it was demonstrated that a man with a bilateral shoulder disarticulation (BSD), who underwent for the first time TMR, on the left side, was able to control a 3 DoFs prosthesis by using sEMG sensors placed on the three muscle sites successfully reinnervated. Prosthesis was composed of open/close (O/C) hand: Greifer Terminal (Ottobock); wrist prono/supination (P/S): Wrist rotator (Ottobock); elbow flexion/extension (F/E): Boston digital arm (included forearm); and, shoulder: LTI-Collier shoulder joint. The proportional control was employed, with a simultaneous joint selection strategy for the hand and the elbow, and with co-contraction strategy to switch between hand and wrist. In details, the patient was able to pass to the control of the wrist from the hand by using the co-contraction of the hand open/close signals; while the elbow was controlled directly with the remaining active site, by modulating the contraction (from weak to strong) for flexing the elbow slightly or completely, respectively; therefore, the elbow extension was possible by relaxing the contraction.

The same author, in [31], also extended the reported outcomes in [21] on a patient with TH amputation, who received the TMR for only two muscle sites. In this case, a powered hand and the same elbow and wrist prostheses presented in [21] were used to simultaneously control three DoFs, by also considering the wrist rotation with shoulder motion.

In Kuiken et al. [24], a case study of a woman with TH amputation was reported to understand if (i) TMR can improve the prosthesis control and (ii) Targeted Sensory Reinnervation (TSR) can provide a region with “sensory perception”, by reinnervating four muscle sites and two sensory sites, respectively. The proportional control with simultaneous selection strategy was used to control a prosthetic device after evaluating, with a grid of 128 monopolar electrodes, the most suitable placements of the sEMG sensors for recording the hand and elbow signals. In this way, the patient was finally able to control a three DoFs prosthesis composed of: a passive shoulder components, motorized elbow (F/E) with a computerised arm controller (LTI), motorized wrist rotator (P/S) (Otto Bock), and motorized hand (O/C) (Otto Bock). To control the wrist, two pressure-sensitive pads were mounted in the patient’s socket, but she rarely operated them because the cognitive load of simultaneously controlling all three joints was high.

In O’Shaughnessy et al. [33], the proportional control technique was used in order to allow a simultaneous control of the prosthesis composed of the elbow F/E and the hand O/C joints. Three TMR patients with TH amputation were enrolled: only two of them had successful nerve transfers and were able to drive the experimental myoelectric prosthesis. For both of the patients, a total of four control sites was used for prosthetic control: the two reinnervated sites to control the hand and two other residual sites for the elbow.

In Miller et al. [34], a case report of three patients with SD and three with TH amputation are presented. The subjects with TH amputation underwent the reinnervation of two muscles, while, for the SD patient, four muscles were reinnervated. All of the patients were equipped with a prosthesis, including Boston Digital Arm, Ottobock device wrist rotator, and electric terminal device. The only difference among the subjects was the use of a prosthetic hand or a hook as a terminal organ. The prosthesis was equipped with proportional control of the elbow and hand joints through the four reinnervated muscle sites in the case of the SD and the two reinnervated sites plus two residual sites in the case of the TH patients. The wrist joint was controlled by the signal from one or two FSR sensors or a potentiometer. At the end of the trials, all of the subjects appreciated the ability to simultaneously control elbow and hand joints, without changing control with respect to conventional control, thanks to the TMR.

To summarize, in all the papers analyzed in this sub-section, the proportional control was employed, combined with both simultaneous and co-contraction joint selection methods. Prosthesis was composed of three DoFs, which were actuated by the different modules that are shown in Figure 5. Only in [33]; the prosthesis was composed of 2 DoFs.

### 5.2. Control via Pattern Recognition

Generally, the pattern recognition strategies applied to the prosthetic control associated the several inputs based on sEMG signals of different movements to several outputs, as limb motions related to specific myoelectric patterns [49].

**Figure 8:**
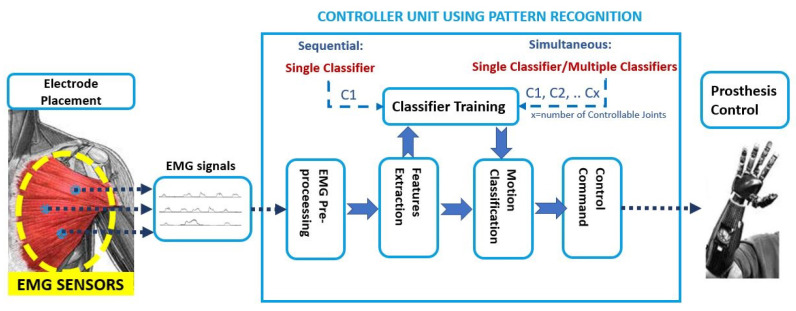
Pattern Recognition approach: the EMG signals are the input to the controller unit. Firstly the pre-processing step is done; then, in the features extraction step, the time and frequency domain features are used as input to train a single classifier or multiple classifiers. The classification output is the motion class to send as the command control to the prosthesis.

These PR algorithms consist of a first step that is based on feature extraction, in the time and frequency domain [50], to enhance information about EMG contraction in selected time windows. Subsequently, in the sequential control technique, a single classifier is trained that is based on linear or non-linear decision boundaries; instead, in the simultaneous control technique, multiple classifiers are trained to control multiple joints simultaneously or a single classifier is trained by considering discrete and combined movements as separate classes, as shown in Figure 8.

For instance, an extensive analysis can be found in [13] and in [51], where a comparative analysis among Non-linear Logistic Regression (NLR), Multi-Layer Perceptron (MLP), Support Vector Machine (SVM), and Linear Discriminant Analysis (LDA) is proposed: the main difference between these algorithms is the linear and nonlinear shape of the decision boundary; straight line or plane for the LDA algorithm; curved line, or surface, for the NLR, MLP, and SVM algorithms. Additionally, the robustness and reliability of the proposed algorithms are key factors for the online control of the prosthetic device and they depend on their offline performance, complexity, and computational time. In the case of trans-radial amputees, the LDA and NLR obtained statistically similar values in terms of F1 Score performance and computational burden in [51].

**Figure 9:**
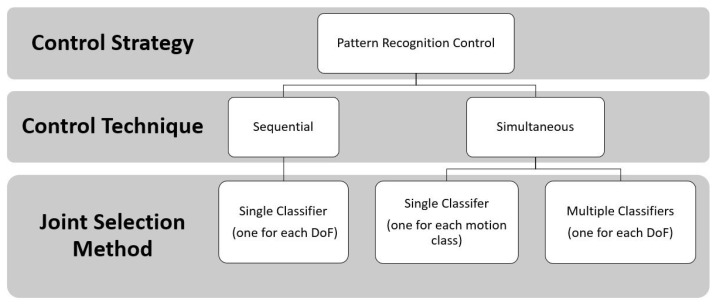
Schematic diagram of pattern recognition-based myoelectric control techniques and joint selection methods.

To sum up, these strategies used machine learning techniques (Figure 9) in order to increase the amputee’s ability to control the prosthesis, in a more natural way, by adding the number of controllable DoFs, because they do not require independent EMG sites for classifying motion classes of different joints [52].

The TMR is considered to be very promising for improving the simultaneous control of multiple arm functions for many (ADLs) ([34,53]). This surgical technique, combined with PR-based systems, represents an opportunity, especially for SD and TH amputees, to overcome the limited number of independent EMG sites that are available for controlling a multi-DoF prosthetic systems [35]. Indeed, the advanced EMG-based pattern recognition strategies have the potential to perform, in a more natural way, the simultaneous control of multiple DoFs with respect to the conventional myoelectric control methods [54], because they do not require independently control sites or mode-switching to activate multiple joints like elbow, wrist, and hand. The following 10 articles have been found in the literature, in which pattern recognition algorithms have been employed in TMR patients:

In this section, for each study, we will analyze the most used strategies introduced to improve the prosthesis myoelectric control for TMR patients with PR control systems, to critically evaluate and compare the performance evaluation results of each method, and to point out the most functional control method and prostheses that replicate the behavior of the human arm.

In Mastinu et al. [28], the monitoring of TMR myoelectric signals of two TH amputee subjects, with TMR surgery and an e-OPRA, has been analyzed for 48 weeks after surgery in order to understand the potentiality as compared to conventional surface electrodes. The TMR-radial and TMR-ulnar sites were used for hand opening and closure, respectively, while the triceps and biceps muscles for the flexion and extension of the elbow. The LDA classifier was used with four TD features: the summation of absolute value of EMG signals, defined as mean absolute value (MAV); the cumulative length of the EMG signal waveform defined as waveform length (WL); the zero crossing (ZC) that measures how many times two consecutive samples have different sign (when the EMG signal crosses zero) in order to detect the onset of movement during the procedure of data segmentation; and, the slope sign changes (SSC), which represents the number of times the slope of EMG signal changes sign. Four discrete motions of elbow and hand were recorded with the Artificial Limb Controller, a prosthetic device that was designed for patients with e-OPRA implants [55].

In Kuiken et al. [35], five TMR patients with SD and TH amputations were able to perform, with a virtual prosthetic arm, 10 different motions that were related to different joints, like elbow, wrist, and hand (elbow F/E, wrist F/E, wrist P/S, hand opening, three types of hand grasps -3 jaw chuck, fine pinch, tool grip, and no movement). For each subject, 12 self-adhesive bipolar EMG electrodes were placed over the reinnervated sites: in detail, four electrodes were placed according to clinical evaluation, while eight additional sites were chosen by an electrode-placement optimization algorithm that allowed to select, from high density (HD) EMG recordings, a reduced number of electrodes necessary to preserve sufficient neural control information for the accurate classification of user’s intention [56]. The proposed PR algorithm was based on an LDA classifier with four TD features (MAV, ZC, WL, SSC). The LDA classifier was used to produce, in real-time, a new prediction every 100 ms. In details, the performance metricsm such as motion selection time (mst), motion completion time (mct), and motion completion rate (mcr), were introduced for assessing the functionality, in real-time, of a virtual multifunction prosthesis.

In Smith et al. [36], the potentiality of PR myoelectric control was investigated when using wireless implantable devices. Five TMR subjects (three with SD and two with TH) were employed for evaluating the capability of performing nine motion classes (rest state, elbow F/E, wrist P/S, F/E, and hand O/C). However, two motion classes (hand open and wrist extension) were excluded for all subjects, because two subjects (one with SD, one with TH) did not have a successful fine-wire insertion into sites. In particular, for two SD subjects, the number of reinnervated muscle sites was equal to 3, while, for one SD subject, was equal to 4. Both intramuscular EMG signals (imEMG) and sEMG signals were acquired by locating bipolar fine-wire electrodes and adhesive bipolar surface electrodes, respectively, on TMR sites. One subject with SD was excluded from pattern classification, because he had the sEMG signals corrupted by a 60 Hz noise.

In Huang et al. [37], different spatial filters were tested in order to enhance the spatial selectivity of EMG recordings and the performance of EMG pattern classification by applying spatial filtering to high-density EMG recordings. Three subjects with TMR were recruited: the first one had a BSD amputation with four reinnervated muscle sites; the second one had a very short TH with four reinnervated muscle sites; and, the last TMR subject had a long TH amputation with two reinnervated muscle sites, and two natively innervated muscle sites. High-density surface EMG signals were recorded from the above mentioned muscle sites, which had been clinically selected. The following fifteen different movements were acquired: elbow F/E, wrist F/E, P/S, ulnar and radial deviation, two hand opening patterns (that included finger abduction and finger adduction), and five functional hand-closing patterns (power grip, prehensile (3-jaw chuck) grip, fine pinch grip, key grip, and trigger grip). The LDA classifier was used to classify the EMG signal with TD features (MAV, ZC, SSC, and WL) and the surface EMG signals were processed by various high pass spatial filters, including one-dimensional and two-dimensional filters.

In Zhou et al. [38], 16 movements of the arm, hand, and finger/thumb, with eight degrees of freedom, were discriminated with an LDA classifier with the TD feature set, and a combination of AR coefficients and RMS (AR-Root Mean Square) of the signals. The recordings were made by using monopolar electrode configuration and three bipolar electrodes in three different directions: transversal, longitudinal, and diagonal. Four TMR subjects were recruited: the first one with a BSD with four reinnervated muscle sites, the second one with a very short TH and four reinnervated muscle sites, and two other subjects with long TH amputations with two reinnervated muscle sites and two natively innervated sites for elbow flexion/extension.

In Batzianoulis et al. [40], three different classification systems that were based on LDA, SVMs (with linear and non-linear kernel), and an Echo State Network (ESN) were evaluated by considering, for each proposed strategy, the classification performance on three phases of dynamic reach-to-grasp motions: acceleration (first phase), deceleration (second phase), and rest (third phase). Eight able-bodied control subjects and four TR amputees, two of which underwent TMR surgery for the neuroma pain, were enrolled. These TMR patients did not have additional muscle sites for improving myoelectric control. The EMG muscle activity was recorded with 12 sEMG sensors from seven muscles of the upper arm and five muscles of the forearm. For LDA and SVM, three features (i.e., average activation of each time window, waveform length, and number of slope changes) for each window of 150 ms have been extracted. Five grasp types (prismatic-2 fingers, precision disk, palm pinch, lateral, and prismatic-4 fingers) were discriminated. In their most recent study [39], the same two TMR transradial amputees that were presented in [40] were employed to extend the previous results by addressing more insights on the LDA potentiality and introducing the use of the Hellinger distance to quantify the similarity between motion classes. In this case, the subjects were asked to perform a bimanual task by only considering three grasp types as the precision disk, lateral, and palm pinch motions. Different from [40], only the performance of an LDA classifier was evaluated in terms of classification accuracy when it was trained for each phase and over all motion phases. To train the classifier, the EMG signals of five muscles of the residual arm were recorded: Flexor Digitorum Superficialis, Extensor Digitorum Communis, Flexor Carpi Ulnaris, Extensor Carpi Ulnaris, and Flexor Carpi Radialis.

In Xu et al. [41], the authors investigated how the rehabilitation training improved the separability of some channels of sEMG signals that remained still coupled over TMR. A TMR TH patient with five targeted muscles with coupled sEMG signals has been engaged. Five bipolar EMG electrodes have been placed on targeted muscles that are associated with the following movements: hand C/O, wrist P/S, and elbow F/E. A new approach that was based on pattern recognition control with MAV-based threshold switches was introduced to improve the classification performance of an LDA classifier, based on Bayesian decision, with TD features (MAV, WL, ZC, and SSC). Subsequently, the obtained classification parameters have been used for allowing the patient to control a commercial prosthesis (Danyang Prostheses Co. Ltd, Danyang City, China); a subset of the modified ARAT test was proposed to compare the online performance of the prosthetic operation.

The LDA classifier with TD-AR (time-domain and auto-regressive) features [57] was introduced for classifying elbow F/E, wrist S/P, and hand O/C. A grid of stainless steel electrodes was placed over specific muscles. However, the exact sites of reinnervated muscles have been not described in detail. The outcome measures, which were obtained with both a virtual reality and a physical prosthetic system, were introduced to evaluate the improvements in terms of offline classification errors. For obtaining physical outcomes, all nine subjects used the following custom-fabricated prosthesis composed of: Boston Digital Elbow (Liberating Technologies Inc.), wrist Rotator (Motion Control Inc.), and single DoF terminal device.

In Hargrove et al. [42], the outcome measures, which were obtained with both virtual reality and a physical prosthetic system, were introduced to evaluate the improvements in terms of offline classification errors of nine transhumeral TMR subjects, when using prosthesis after a six-week home trial. Three blocks of the Target Achievement Control (TAC) test [58] were used to evaluate the performance of the LDA classifier with TD-AR (time-domain and auto-regressive) features [57]. For obtaining physical outcomes, all nine subjects used the following custom-fabricated prosthesis that was composed of: Boston Digital Elbow (Liberating Technologies Inc.), wrist Rotator (Motion Control Inc.), and single DoF terminal device.

In Tkach et al. [43], it was demonstrated that a generic grid arrangement of electrodes performed equivalently or better than the control site (specific site for electrode placement). Four TMR amputee subjects were employed: two TH subjects had four reinnervated muscle sites; two SD subjects presented, instead, only two reinnervated muscles sites. EMG signals were acquired by using 15 bipolar pairs of EMG electrodes placed according to two conditions: in the “Control Site” condition, the electrodes were placed over muscle control sites, after clinical palpation; in the “Grid” condition, electrodes were positioned in a grid configuration, around the residual limb and the surface of the chest, for the TH and SD subjects, respectively. The LDA algorithm was used with the AR feature set, including the six coefficients of a 6th order autoregressive model.

To sum up, all 10 studies presented in this sub-section take the pattern recognition strategy based on LDA classifier with different features set into account: TD features (MAV, WL, ZC, and SSC) [28,35,36,37,38,41]; TD-AR features [57]; AR-RMS [38]; the AR feature set [43]; Hellinger distance [39]; and, the average activation of each time window, the waveform length, and the number of slope changes [40]. In Batzianoulis et al. [40], the SVMs (with linear and non-linear kernel), and an Echo State Network (ESN) PR-based strategies were also evaluated by considering, for each proposed strategy, the classification performance on five reach-to-grasp motions. The minimum number of discriminated classes was equals to four discrete motions related to the elbow and hand [28] or only the hand [39,40]. While, for the other seven studies, the elbow, wrist, and hand joints were always considered by including from nine up to 29 motion classes [43] (for both discrete and simultaneous movements).

### 5.3. Comparison between DC and PR Strategies

The following four papers presented a comparison between direct control and pattern recognition based strategies.

Particular attention was paid to information regarding the level of amputation, the number of sites reinnervated and used for the control, the various DC strategies and PR algorithms used, and the prosthetic device used (when indicated).

The first study that directly compared the performance of pattern recognition systems to direct control systems using a physical prosthesis with TMR patients is Hargrove et al. [44]. Four patients (one male with SD, two males and one female with TH amputation) had at least four reinnervated control sites (five in one case) used for direct control of the elbow F/E and hand O/C joints. The P/S of the wrist joint was controlled and selected in different ways by the various patients, in a manner similar to that used with their old prostheses. For the PR-based control system, four pairs of bipolar electrodes have been added to the four pairs that were used for direct control. The PR control system was composed of a LDA classifier with TD features and AR coefficients. The velocity of the desired movement was computed while using a simple proportional control algorithm. Section 6.3 presents the performance achieved by all patients experts in the daily use of the myoelectric prosthesis with DC control and with experience in the laboratory use of the prosthesis controlled with PR. All of the subjects said that they preferred PR-based control, because it was more intuitive. However, the authors pointed out that direct control allowed the simultaneous movement of two joints, while the PR-based control was limited to sequential control, even when tasks required multiple DoFs.

In Wurth et al. [45], a real-time comparison between DC and PR-based control strategies was carried out to control a multi-DoF myoelectric prosthesis. Only one TH amputee among the enrolled subjects underwent the TMR procedure, with four independent control sites. The others were nine healthy control subjects and one TR amputee. For the DC control, the MAV EMG signals of the wrist flexors and extensors muscles were recorded from able bodied and TR subjects by using pre-gelled adhesive bipolar Ag-AgCl electrodes. Instead, for the TH amputee subject, four bipolar electrodes were placed on the flexor and extensor muscles, in order to simultaneously control more than one DoF. For the PR-control, the LDA classifier was used with four TD features (MAV, ZC, SSC, and WL) and six AR coefficients. In particular, the able bodied and TH subjects were asked to perform hand O/C, wrist F/E, and no motion. Instead, for the TH subject, the elbow F/E was replaced by wrist F/E, because this DoF was considered to be more intuitive and relevant to be controlled for this level of amputation.

In Hargrove et al. [46], a clinical study was reported on eight TH patients, with different levels of amputation and prosthetic solution composed of motorized Boston Digital Elbow (LTI), Motion Control Wrist Rotator (Motion Control Inc.), and terminal device (seven hook from Greifer or EDT and one hand). All of the subjects used prosthesis in both the controlled (laboratory) and uncontrolled (home) environment. The eight patients were randomly divided into two groups of four subjects, each of which completed the home-trial while initially using a prosthesis with a different control strategy, according to the group that they belonged to. The two configurations were used for six weeks each. The electrode sites were identified with different methods, depending on the different strategy adopted: when using direct control, the muscle sites were manually udentified, using a combination of surgical notes when available, palpation, and myoelectric signal testing. As for the PR control, linear electrode locations were not targeted over specific muscles, rather a grid of electrodes was used. The algorithm that was used for PR-based control was the LDA described in [57]. For the DC control, dual-site differential DC system employed antagonistic muscle pair in order to control elbow F/E and terminal device O/C (hand or hook). In addition, mode switches were configured, for each subject, to control the wrist P/S DoF according to their previous device use.

In Young et al. [47], three different control strategies (direct control with proportional strategy, sequential PR control—one DoF at time, and simultaneous PR control—two DoFs at time) were analyzed in order to evaluate the ability of four amputees (two TH and two SD), who underwent TMR surgery, to simultaneously control up to two DoFs with a virtual prosthesis. TH patients had two reinnervated muscle sites used for controlling hand O/C movements, and two natively innervated muscles (the biceps and triceps brachii) used for elbow F/E motions. The SD subjects had four reinnervated sites for controlling hand O/C and elbow F/E movements. Four pairs of self-adhesive Ag/AgCl bipolar surface electrodes were placed in the same muscle sites that were used for the conventional prostheses control. Other pairs of electrodes were placed near the primary sites where muscle activity could be palpated. The following eight discrete and combined motions were acquired: elbow F/E, hand O/C, and elbow F/E combined with hand O/C. The TMR amputees controlled discrete motions using their four independent muscle sites. For the PR-strategy, an LDA algorithm with four TD features (MAV, ZC, SSC, and WL) and six AR coefficients of a sixth-order were used for the classification. As for the sequential control condition, the same methods that were introduced in [48] were used. Instead, for the simultaneous control strategy, the authors used the system that was tested on able-bodied subjects in [59].

To summarize, in all of the articles presented this section, a physical prosthetic device with two [45] or three DoFs [44,46] was employed, except for the [47], in which a virtual prosthesis was used instead of the physical one. Regarding the direct control strategy, in all of the studies, the simultaneous joint selection methods were employed. Only in Young et al. [47] was the control technique specified, i.e., the proportional control technique. Regarding the reviewed papers on PR-based control, the LDA was always adopted with TD and AR features.

## 6. Performance Evaluation Methods

In this section, the performance evaluation methods introduced in the analyzed papers are reported. These methods allow for evaluating the functional effectiveness of the prosthesis as a whole with quantitative indicators that highlight the potential and advantages of the proposed systems.

The reported studies have shown the use of many performance evaluation criteria, although there is no standard procedure in place so far. The lack of a common standard is highlighted by the presence of multiple criteria that are necessary for the performance evaluation in the analyzed papers. In Table 2, for each method, the following information are reported: the percentage of the number of patients enrolled with respect to the total number of the patients considered in all the papers (i.e., 67, which is the sum of column 2 of Table 1) and, the total number of articles in which the performance evaluation method was employed.

### 6.1. Direct Control

Regarding systems with DC strategies, the following performance evaluation methods were taken into account: in the Box and Block Test (BBT) [60], the patient had to move the higher number of standard size cubes from one side of the box to the other in a maximum time of one minute; however, Kuiken modified the test extending the time limit up to 2 min., to use it with amputee patients who had undergone the TMR procedure. In the Clothespin Relocation Test (CRT) [21], the patient is prompted to move three clips from the horizontal bar to the vertical bar; the execution time that is required to complete the task was measured. The test was repeated three times. Instead, the Cubbies test [32] is a cubicle reach and retrieve test (Cubbies) composed of 15 cubicles, containing 15 one-inch (2.5 cm) cubes placed on an adjustable height table; in this test, the subject, without moving his feet, has to reach, grasp, and place the 1-inch cubes on the table from as many cubicles as he is able to reach successfully; the total time to grasp a 1-inch cube, place it on the table, and activate a buzzer is recorded. The score consists of the average time per successful reach and retrieval of all blocks in the work-space. In the Cups test [32], 11 plastic cups must be individually retrieved from an inverted stack and positioned in a prescribed pyramid arrangement; four upside-down cups on the bottom row, until one on the top. The final eleventh cup has to be placed upright on top of the top upside-down cup of the pyramid. The time that is needed to stack and unstack the cups is recorded. Finally, the Miscellaneous ADL consisted of a series of the following activities: cutting meat with a knife and fork; place three objects onto a tray and then transport the tray; place 3 1-lb cans into a bag with handles; open and close a jar of peanut butter; stir a spatula in a big bowl; open an envelope with a tool; wrap a package; pull on both socks; and, remove and put on a long-sleeved shirt.

The following six papers used the BBT (modified up to 2 min.) [21,24,31,32,33,34] to evaluate the performance of the proposed control strategy. In detail, in Kuiken et al. [21], the comparison of the prosthetic control before and after the TMR surgery has shown an improvement of the new procedure. An increase in performance was present: +8.3 blocks, on average, over three tests. In Kuiken et al. [31], for the patient with TH amputation, the results were: +322% blocks moved with the experimental prosthesis. In Kuiken et al. [24], there was the evidence of an increase in the performance between pre-surgery and post-surgery: the average score over three trials changed from 4.0 ± 1.0 to 15.6 ± 1.5 blocks. In Miller et al. [32,34] and O’Shaughnessy et al. [33] the BBT (modified up to 2 min) was employed. In Miller et al. [32], the patient achieved better performance with the three DoFs arm: he moved, on average, 15.0 ± 0.1 blocks, while wearing the six DoFs prosthesis he only moved 13.7 ± 2.5 blocks. In Miller et al. [34], the improvements ranged from 95% to 271% (with an average of 198%) of the number of blocks moved. In O’Shaughnessy et al. [33], both of the patients demonstrated an increase in the number of blocks (on average, over three tests) of 611% and 150% for patient 1 and patient 2, respectively.

The CRT was also employed in the following six studies [21,22,31,32,33,34]. The task execution time of CRT equals -36 seconds in Kuiken et al. [21,22] and −54.24% in Kuiken et al. [31]. In Miller et al. [32], the subject was required to flex the shoulder forward once, and then sequentially activate terminal device, elbow and wrist rotations. This test showed that the subject was faster with the six DoF arm (58.0 ± 9.2 s of task execution time) than the three DoF arm (79.2 ± 14.3 s of task execution time). In Miller et al. [34], the improvement ranged from 31% to 55% (with an average of 45%) reduction in the time to complete the task. In O’Shaughnessy et al. [33], there was a decrease of the time (in seconds) that is required to move three clothespins from one beam to another: 55% and 41% for patient 1 and for patient 2, respectively.

The AMPS test was employed in the following four papers [24,31,33,34]. In Kuiken et al. [24], it went from the score of 0.30 and 0.90 for motor and process, respectively, to the score of 1.98 in both cases. In O’Shaughnessy et al. [33], a computer-tabulated score was reported, reflecting motor and processing function concerning activities of daily living. For patient 1, there was an increase in the score from 0.5 to 1 regarding the motor function and from 0.3 to 1.1 in the processing function. For patient 2, there was an increase in the score from 0.9 to 1.56 in the motor function and from 1.09 to 1.43 in the processing function.

In Kuiken et al. [31], the Wolf Motor Functions Tests (WMFT) [61] was also performed in addition to the Assessment of Motor and Process Skills (AMPS) [62]. The patient with TH amputation only had an increase in the score of +0.54 and +0.75, respectively. Thus, for the TH patient, a markedly greater increase in performance has been demonstrated than that obtained by the BSD patient. In Miller et al. [34], the tasks included cooking, cleaning, housework, garden work, and home maintenance; all of the TH subjects and two of the three SD amputees performed the AMPS test. For the SD amputees, an average increase in the motor score of +0.80 occurred; instead, for the TH subjects, the average scores was equal to +0.77; for the process score, there was an average score increase of +0.5 for the SD amputees, and +0.57 for TH ones. However, it should be pointed out that, as the current tests may not adequately measure improvements in control or prosthetic design due to the ceiling effect (the task is too easy to be performed by most subjects) or to the floor effect (the task is too difficult, almost none of the subjects can perform it).

Only in O’Shaughnessy et al. [33], the Miscellaneous ADL was evaluated: both of the patients used less time to complete the various activities (except wrapping a package) when using the experimental prosthesis.

Finally, in Miller et al. [32], the Range Of Motion (ROM), the Cubbies, and Cup test were also employed. In detail, an increase of the ROM of the shoulder flexion: it went from a range 0–90${}^{\circ }$ for the passive shoulder with the old prosthesis to a range of 200${}^{\circ }$, from −15${}^{\circ }$ to +185${}^{\circ }$, with the new experimental prosthesis. Regarding the Cubbies test, the ROM was greater with the six DoF device due to the shoulder and wrist motors: with the three DoF prosthesis, the subject was only able to access 12 of the cubbies, whereas, with the 6 DoF device, the patient was able to access all 15 cubbies. Even with the increased work-space, the time per cubby was approximately the same: 18.6 ± 2.5 s with the 3 DoF arm and 15.3 ± 9.5 s. In the Cups test, the subject showed an increase in the amount of time necessary to stack all 11 cups in an inverted pyramid using the 6 DoFs (357 ± 36 s), compared to the 3 DoFs prosthesis (169 ± 58 s). To unstacking cups, the difference was lower: (83 ± 13 s) with the three DoF prosthesis and (89 ± 29 s) with the six DoFs one. Interestingly, the six DoF prosthesis was not always better than the three DoF prosthesis, on the contrary, it was sometimes worse due to some reasons as: the considerable weight (about 5.75 kg) of the six DoF prosthesis and the patient’s cognitive effort forcing him to perform the tasks more slowly. Thus, to make a six DoF limb prosthesis clinically usable, it is necessary to develop a simpler and more intuitive control system, avoiding using the shoulder to control wrist movements.

### 6.2. Control via Pattern Recognition

Instead, in systems with PR control strategies, the following performance evaluation methods were taken into account: the offline classification accuracy [28,35,37,38], the offline classification error [28,36,42,43], the mst, mct, and mcr [35], the mean path length error percentages [43], and the score of six tasks rhat were chosen from Action Research Arm Test (ARAT) [41].

In detail, in Kuiken et al. [35] the performance metrics as the mst, mct and mcr were introduced for evaluating the control of a virtual arm in real-time, even if that of a real prosthesis is more challenging. In detail, mst was defined as the time from the onset to the first correct classification (i.e., the time that is taken to successfully select a target movement); mct was the time from movement onset to the 10th correct classification (i.e., the time from the onset to the completion of the intended movement); finally, mcr (“success” rate) was the percentage of successfully completed motions out of the total attempted motions.

In Xu et al. [41], the following six grasping tasks from the ARAT test were performed for assessing the recovery of upper limb function [63]: the grasping of a Block of 2.5, 5.0, and 7.5 cm^3^, of a cricket ball, and a sharpening stone; to pour water from one glass to another; to displace 1 and 2.25 cm alloy tubes from one side of the table to another.

Only in Hargrove et al. [42], the following TAC, SHAP, JTHFT, and ACMC tests and the virtual outcome metrics were employed in order to evaluate the performance of the PR system as following. In particular, three blocks of Target Achievement Control (TAC) test [58] consisted of moving, within a 15-s time frame, a flesh-colored virtual limb to match the 12 postures of a translucent grey-colored virtual target limb % in real-time. For the virtual outcome metrics, the number of postures successfully acquired within their allotted 15-s time frames, and the median completion time required to match the set of postures in a block, were analyzed. Instead, for evaluating the differences between the control of a virtual prosthesis in a virtual environment with respect to that of the physical prosthesis, the following parameters were introduced as physical outcomes: the classification error rate, the completion time, and the failure rate. The classification error rate is defined as the number of incorrect decisions that were divided by the total number of decisions; the completion time was the time from the trial start to the target posture achievement, while the failure rate measured the percentage of trials that were unsuccessfully completed during the TAC tests. These physical outcomes were evaluated by using the following promising tests for assessing the functional effectiveness of a prosthetic system [64]: the Southampton Hand Assessment Procedure (SHAP) test [65], which consists of 12 abstract objects and 14 ADLs, and each task is timed by the participant in order to avoid reliability on the reaction times; Jebsen–Taylor test of Hand Function (JTHFT) [66], which is made up of seven subsets that are writing, simulated page-turning, lifting small objects, simulated feeding, stacking, and lifting large, lightweight, and heavy objects; the Assessment for Capacity of Myoelectric Control (ACMC) [67], which consists of 22 items that are related to capacity for myoelectric control: the need for external support, grip force, coordination of both hands, different positions and in motion (timing), repetitive grasp and release, and the need for visual feedback. It is the only test validated for a good test-retest reliability and interpretation guidelines for evaluating the functionality of upper-limb prostheses [68]; and, BBT and CRT (as described in Section 5).

The following six papers used the classification errors [28,35,37,38,39,40] to evaluate the performance of the proposed PR-based control strategy.

In Mastinu et al. [28], four movements that were related to elbow and hand were discriminated with a sequential pattern recognition strategy based on a LDA classifier with TD features. The offline classification accuracy was over 97% since the last follow-up (week 48). Their results showed, for the first time, the evolution and quality of the TMR signals when using intramuscular electrodes instead of the conventional skin surface electrodes. In Kuiken et al. [35], ten different motions of elbow, wrist, and hand were classified with a sequential PR strategy that is based on a single LDA classifier with TD features. The mean classification accuracy was 88 ± 7% for TMR patients. In Huang et al. [37], fifteen different discrete movements were discriminated by using a single LDA classifier with TD features (MAV, ZC, SSC, and WL). The surface EMG signals were processed by various high pass spatial filters, including one-dimensional and two-dimensional filters. The use of high-density EMG recordings combined with a single differential filter in transverse direction (BipT), and a single differential filter in longitudinal direction (BipL), or higher order filters, allowed for reaching 95% classification accuracy for 15 movements for SD and patients with a long transhumeral amputation, and above 85% for patient with a short transhumeral amputation. However, when only considering 12 EMG signals, the double differential filters obtained 5–15% higher classification accuracies than the filters with a lower spatial resolution and comparable accuracies to the filters with higher spatial resolution. Thus, the use of double differential EMG recordings can improve the TMR-based neural interface for the control of artificial arms. In Zhou et al. [38], a single LDA classifier with the TD feature set, and a combination of AR coefficients and RMS (AR-RMS) of the signals were used to classify 16 movements of the arm, hand and fingers. The performance of the LDA classifier was reported in terms of classification accuracy for the various electrode configurations: with the monopolar channels, the average overall classification accuracy was equal to 90.5 ± 6.3% for TD feature sets and 90.0 ± 7.3% for AR-RMS feature sets. The accuracy of classification consistently improved to an average of 96.0 ± 3.9% with TD and to 95.0 ± 5.2% with AR-RMS features, for bipolar electrode configurations. Thus, TMR combined with the LDA classifier was able to extract motor control information from the reinnervated sites, by using high-density surface EMG recordings. In Batzianoulis et al. [40], a comparison of offline classification accuracy of four different classification systems based on LDA, two SVMs, and an Echo State Network (ESN) was presented. By applying an analysis of variance (ANOVA) (with a significance level of 5%) to the classifiers’ performance, a significant difference for all the proposed classifiers in terms of offline accuracy values, for all 5, 4 and 3 classes of movements was not found. Namely, in the first and third phase, the average classification accuracies are similar for both non-TMR and TMR patients: 68.6 ± 8.8% and 64 ± 14.4% for the first phase and 87.6 ± 3.4% and 83.6 ± 4% for the third phase, respectively. However, the accuracy of TMR subjects in the second phase was better than that of non-TMR subjects (90.2 ± 4.6% and 77.8 ± 10.9%, respectively). Finally, an on-line evaluation of 20 reach-to-grasp motion with RIC hand [69] was tested only for a non-TMR subject. In Batzianoulis et al. [39], the EMG signals were analyzed by applying a sliding time window of 150 ms with an increment of 50 ms and three features (MAV, SSC, WL) were extracted from each time window. The classification system was composed by three LDA classifiers, one for each phase: the accuracies values were equal to 42.7 ± 8.2%, 57.8 ± 14.4%, and 74.2 ± 14% in the first, second, and third phase, respectively. Instead, the single LDA classifier, which was trained with all the phases, obtained an accuracy of 33.6 ± 12.5%, 51 ± 15.4%, and 66.2 ± 11% for each phase. The obtained results underlined that the arm extension towards a specific direction during a reach-to-grasp motion affected the classification performance. Thus, the introduction of segmentation into motion phases revealed that higher accuracy values can be obtained when considering all of the reach-to-grasp motion phases.

The following four papers used the classification errors [28,36,42,43] to evaluate the performance of the proposed PR-based control strategy.

In Mastinu et al. [28], the evolution of the classification error over time was considered: for the first subject, the mean error decreased from 10.8% (week 4) to 1.7% (week 48), while, for the second subject, it remained stable below 5%. In Smith et al. [36], two LDA classifiers with TD [48] and autoregressive (AR) features [56] were implemented, by using sEMG and imEMG signals as input, respectively. The use of imEMG instead of sEMG produced a decrease of 1.39 ± 6.45% (90% confidence interval) of the average error rate, which was equal to 5.52% for sEMG and 4.13% for imEMG. The proposed results showed that, despite the variability of imEMG signals, the performance of the LDA classifier did not decrease. Thus, the imEMG signals can also be used for the PR myoelectric control, with the benefits of the reduction of EMG crosstalk, the placement on deeper muscles, and the overcoming of electrode shifting. However, the presence of sparse motor units at the reinnervated sites increased the MAV, based on the amplitude of the imEMG signals, and, consequently, this feature could not be used for estimating the proportional velocity. In Hargrove et al. [42], the average classification error across subjects decreased from 13.4 to 8.3% after home trials. In Tkach et al. [43], the classification errors of five classifiers, based on the LDA algorithm with the AR feature set, were evaluated when two different electrode placements were considered: the “Control Site” and Grid “configurations” (as described in Section 5.2). The five classifiers differ from the output classes: the “Seq Only” PR strategy was based on a single classifier based on 9 motion classes; the “Seq. Elbow+Hand”, the “Seq. Elbow+Wrist”, the “Seq. Hand+Wrist”, and “All” employed a single classifier that was able to classify 13,17,17 and 29 motion classes, respectively. In detail, the eight discrete and twenty combined motions were the following: elbow F/E, wrist F/E, wrist P/S, and hand O/C; elbow F/E + hand O/C; elbow F/E + wrist F/E; elbow F/E + wrist P/S; wrist F/E + hand O/C; wrist P/S + hand O/C; no motion condition. The discrepancies of the classification error between the Control Site and Grid conditions were equals to 11.47% for the “All” classifier (29 motion classes) and 1.69% for the “Seq Only” classifier. The “Seq. Elbow+Wrist” and the “Seq. Hand+Wrist” classifiers, which discriminated 17 motion classes, both had similar classification errors of 11.5 ± 1.1% and 11.2 ± 1.2% for the Grid analysis condition. Regarding the Control Site condition, the two classifiers yielded higher errors of 17.9 ± 1.3% and 21.1 ± 1.7%, respectively. These results showed that the grid-like arrangement of electrodes can outperform the specific electrode placement on targeted muscle sites when considering classifiers with a greater number of motion classes.

For the real-time evaluation, in Kuken et al. [35], the LDA classifier was also used to produce, in real-time, a new prediction every 100 ms. The mean motion completion rate for the elbow and wrist movements was high (96.3 ± 3.8%) and it was lower for hand grasp movements (86.9 ± 13.9%) that were considered to be more challenging by some patients. Thus, the relevance of this study was to have assessed, for the first time, a protocol to evaluate, in real-time, the PR performance for controlling multi-DoF artificial arms, in patients with TMR.

Only in Xu et al. [41], comparisons have been made between the PR algorithms with no post-processing (Control), majority vote, and MAV-based threshold switches, while using six tasks that were chosen from Action Research Arm Test (ARAT). An LDA classifier based on Bayesian decision has been adopted with TD features (MAV, WL, ZC, SSC). The final scores were reported for the three methods: for PR control, they were equal to 11, 11, and 13 with majority vote lengths equal to 3, 5, and 10, respectively; for MAV-based threshold switches, three threshold values were defined: the standard threshold (ST) was equal to 0.2 mV; the lower threshold (LT) was equal to the 80% of ST; and, the higher threshold (HT) was equal to the 120% of ST. The final score values were equal to 14.7 (with lower threshold), 16 (with medium threshold), and 14.7 (with higher threshold). Finally, the improved PR control with MAV-based threshold switches turned out to be the best configuration for obtaining a robust control of the prosthesis.

Only in Hargrove et al. [42] were the following results about these performance evaluation tests reported: the TAC test performance metrics improved significantly from 19.9 to 3.7% after home trials: the failure rate improved from 19.9 to 3.7% (*p* = 0.001), and the completion time decreased from 7.5 to 5.5 s (*p* = 0.007). In the virtual test, the median completion time correlated significantly with the Southampton Hand Assessment Procedure (*p* = 0.05, R = −0.86), Box and Blocks Test (*p* = 0.007, R = −0.82), Jebsen–Taylor Test (*p* = 0.003, R = 0.87), and the Assessment of Capacity for Myoelectric Control (*p* = 0.005, R = −0.85). The classification error performance only had a strong correlation with the Clothespin Relocation Test (*p* = 0.018, R = 0.76). However, only the SHAP (*p* = 0.001) and the BBT (*p* = 0.03) have showed statistically significant improvements after a six-week home trial. Additionally, the physical outcomes that were related to the use of the physical prosthesis improved after the home trial. Thus, when considering all metrics as the classification error rates, the outcome metrics associated with both the virtual TAC test and the physical prosthesis, the home trial is the best solution for make subjects able to control the device.

Finally, in Tkach et al. [43], the mean path length error percentages and the mean offline classification errors of classifiers, with lower complexity than those reported above for the offline evaluation, were also considered with a virtual limb, for both experimental conditions (“Control Site” and “Grid”): the sequential real-time classifier (“SeqRT”) was trained to predict four single-joint motions (elbow F/E and hand O/C, and no motion class); the simultaneous real-time classifier (“SimRT”) was trained to predict the no motion class, the four single-joint motions of “SeqRT”, and four combined motions classes (elbow F + hand O; elbow F + hand C; elbow E + hand O; and, elbow E + hand C). In particular, as regards the “SeqRT” classifier, the mean path length error percentages for “Control Sites” and “Grid” conditions were equal to 68.25% and 68.99%, while, for the “SimRT” classifier, they equaled 22.48% and 25.25%, respectively. Instead, the mean offline classification errors were equal to 1.6% (“Control Sites”) and 1.3% (“Grid”) for the “SeqRT” classifier; as regards the “SimRT” classifier, the mean offline classification errors were equals to 19.2% (“Control Sites”) and 17.1% (“Grid”). The real-time results demonstrated that the simultaneous PR control of multiple DoFs perform equivalent or slightly better by using either a grid arrangement of electrodes or site-specific electrode placement.

### 6.3. Comparison between DC and PR Strategies

Finally, in the papers that reported a comparison between DC and PR, the performance evaluation methods were the following.

In Hargrove et al. [44], the performance of the proportional DC control and LDA classifier with TD features and AR coefficients was compared. The patients completed three different real-time performance tests with each system: BBT (modified up to 2 min) and block stacking test [70] (this test involves stacking the largest number of 1-inch cubes on top of each other in three minutes), CRT. In the BBT, the patients achieved an average 40% increase in the number of blocks passing from direct to PR control; in the block stacking test, the stacked towers were 59% higher while using the pattern recognition control system. The clothespin task was completed in 25% less time when using the pattern recognition control system. The average classification error rate for the pattern recognition systems was 16.3% (±1.6%).

In Wurth et al. [45], the real-time comparison between DC and PR-based control strategies have led to the following performance outcomes. In detail, the authors developed the Fitts’ target acquisition task (FTAT) test, based on Fitts’ law, which consisted of moving a cursor in two-dimensional Cartesian space from the center of the screen to a circular target appearing within a given radius at a given position. It was used in real time for assessing three EMG-based control strategies in virtual environments: the clinical standard of care (DC), a conventional, sequential PR (seqPR) strategy, and a simultaneous PR (simPR) strategy. The simPR approach considered the classification of each DoF independently. The parallel strategy that was introduced by [71] was adopted for the simultaneous classification of both DoFs. In order to comprehensively compare the three strategies, the following principal functional performance metrics were introduced: throughput (bits/second) and path efficiency (%). In detail, the throughput values for discrete motions (1 DoF) were equal to 2.64 ± 0.24, 3.67 ± 0.23, and 2.11 ± 0.18 for the conventional, seqPR, and simPR, respectively. Instead, for the combined motions (two DoFs), the throughput values were equal to 1.24 ± 0.04, 1.32 ± 0.03, and 1.63 ± 0.05 for the conventional, seqPR, and simPR strategies, respectively. Regarding the path efficiency values, they were equal for discrete motions (1 DoF) to 90.1 ± 0.23, 97.0 ± 0.96, and 96.3 ± 1.12 for the conventional, seqPR, and simPR strategies, respectively. Instead, for the combined motions (two DoFs), the path efficiency values were equal to 71.3 ± 0.8, 71.6 ± 0.76, and 87.7 ± 0.7 for the conventional, seqPR, and simPR strategies, respectively. Regarding discrete one DoF motions, the functional performance metrics of simultaneous PR were slightly lower than the sequential pattern recognition strategy that was revealed to be more precise and robust. Additionally, a qualitative evaluation of the control strategies was performed through a questionnaire demonstrating that both pattern recognition control strategies outperformed the amplitude-based DC control when considering two DoF tasks. In fact, the DC control was felt as unnatural and cumbersome in operating two 2 DoF control. It was only considered to be efficient for discrete 1 DoF tasks.

For the clinical trial reported by Hargrove et al. [46], the performance evaluation was carried out, for each type of control (PR and DC), by carrying out a series of tests before and after the six weeks of the home-trial: ACMC, SHAP, BBT (modified up to 2 min), and CRT. At the end of the trial, there were no significant differences in the ACMC test scores between pattern recognition (47.3 ± 3.9) and direct control (44.4 ± 3.4). As for the SHAP test, a significant improvement (*p* = 0.041) was noted in the performance that was achieved by using the PR-based control as compared to the direct control. The authors also noted that there was a difference between the performance in the pre and post trial cases (*p* = 0.038). In the BBT test, there was no particular difference between the pre and post trial tests; however, it was noted that, at the end of the home trial, the subjects moved 13.4 ± 2.6 blocks using pattern recognition control and 15.6 ± 2.7 blocks using direct control. Finally, in the CRT test, the subjects obtained significantly better results (*p* = 0.024) (i.e., the patients needed less time for concluding the test) using the PR-based control (90.2 ± 39.6 s) than using the direct control (137 ± 60.2 s). There were no statistically significant changes between pre- and post-home tests, nor was there any significant interaction between the pre- and post-home tests and the control strategy used. Quantitative statistics on household use have been carried out, as measured by the control system: on average, users cumulatively wore the prosthesis 138.7 ± 34.6 h during the direct control portion of the home trial and 147.7 ± 45.3 h during the PR phase of the home trial. The subjects chose to re-calibrate their control on 32.6 ± 8.2 occasions over the duration of the home trial. At the end of the trial, seven out of eight patients said that they preferred PR-based control over DC. As for DC, many had found pulse control not intuitive; they also found it difficult to control a single DoF when desired, having the simultaneous activation of two DoFs available. On the other hand, in the PR-based control, the subjects reported that the prosthesis was sometimes activated unwantedly.

In Young et al. [47], the performance of the conventional amplitude-based myoelectric control, the sequential (one DoF at time) PR control, and the simultaneous PR control (two DoFs at time) were reported. That three strategies were evaluated with a virtual prosthesis in a virtual environment using the TAC Test that considered the completion time, completion rate, and length error as performance metrics. The statistical ANOVA test (p<0.05) was conducted for the comparison of the reported control strategies. For two DoFs tasks, the simultaneous PR system performed the best, with the lowest average completion times, completion rates, and length error when compared to the other control strategies. In particular, for the 2 DoFs tasks, the amputees chose to perform simultaneous movements in 78% of cases with simultaneous PR and in 64% of cases with conventional control. Furthermore, overall offline classification errors for the PR control strategies were compared with the ANOVA test (p<0.05). The average classification error for sequential control was equal to 11.1% (±5.8 standard error of the mean—SEM), while the errors for simultaneous PR control were 23.1% (±10.3 SEM) and 33.19% (±11.3 SEM) for discrete and combined movement classification, respectively. Thus, finally, the authors have demonstrated that the simultaneous PR system had slightly lower performance with respect to the sequential PR system that was used for 1 DoF tasks (that required one DoF motions) and performed better than conventional control on 1 DoF tasks, while it had the best performance with simultaneous PR control on 2 DoFs tasks when compared to both conventional or sequential control.

Table 3 summarizes the results of the comparison papers between DC and PR. In most cases, the systems that are based on PR strategy allow for achieving better performance than DC one.

## 7. Results and Discussion

A comprehensive literature analysis on the most used prosthetic control strategies for TMR patients was carried out, when considering the amputation level of the enrolled patients, the number of signals of reinnervated sites, the number of controlled DoFs and, when available, the methods for validating the control of a prosthetic device, and the obtained performance. When the prosthesis is controlled with DC strategies (proportional strategy, usually), it is possible to use the simultaneous method for the selection of the joint to be controlled. The number of joints that can be controlled simultaneously depends on the number of input signals available. The reinnervated sites used to acquire EMG signals are usually three [21] or four [31,32,34,44,47] for SD patient, while it can be two [31,33,34,47] or four [24,44,45] for TH amputees. Especially when there are less then two reinnervated sites, additional signals from the residual muscles [21,31,33] can be used to actuate the prosthesis with the myoelectric control. However, patients simultaneously control, at most, two DoFs with the only use of EMG signals, whether from reinnervated sites or residual muscle. Almost all patients have demonstrated that they can control a three DoF prosthesis with F/E elbow, P/S wrist, and O/C hand movements, by switching with the co-contraction joint selection strategy from one of the two DoFs simultaneously controlled (usually elbow or hand) to the third one (wrist) and vice-versa. For patients with more than four reinnervated sites, as one of the TH amputees in [44], the P/S of the wrist can also be controlled simultaneously. In some studies, additional inputs were added to EMG signals, such as those from FSR sensors [32,34] or from switches [24]; these additional inputs allow for simultaneously controlling more than two DoFs, with a hybrid control. However, prostheses with more than three DoFs, as in [32], resulted in being difficult and less intuitive to control.

With respect to the conventional myoelectric control methods, which only consider the EMG amplitude at specific myoelectric control sites [54], the PR control systems that are based on both sequential and simultaneous strategies allow for the control of up to 2 DoFs, in a natural way, as asserted in [44,46], and [45,47]. Regarding the PR strategies, most studies considered the following discrete motion classes most clinically used for different amputation levels. In the case of BSD, SD, and TH amputees, from eight to 16 motion classes were mainly considered: elbow F/E, wrist F/E, wrist S/P, wrist ulnar and radial deviation, hand opening, two hand opening patterns, including finger abduction and finger adduction, a selection of various types of hand grasps (as 3-jaw chuck, power grip, fine pinch, key grip, trigger grip, and tool grip), and a rest state [35,36,37,38]. Instead, for the TR TMR amputees, as considered in [39,40], from three to a maximum number of five grasp types were taken into account: prismatic-2 fingers, precision disk, palm pinch, lateral, and prismatic-4 fingers. The majority of these studies [36,37,38,39,40] reported only the offline performance of the LDA classifier with TD, AR, AR-RMS, and TDAR, except [35], which introduced useful performance metrics as motions selection time, motion completion time, and motion completion rate for evaluating the control of a virtual arm in real-time. When only considering that the offline accuracy can be a relevant limitation for evaluating the prosthetic control, since many studies [72,73] have shown that offline accuracy does not necessarily correspond to real-time performance. To overcome these limitations, other studies, such as [41,42], introduced for TH amputees a physical prosthesis and a virtual limb for controlling, using an LDA classifier with time-domain feature set, the following discrete movements: elbow F/E, wrist rotation, and hand C/O. It is worth noticing that, in [42], the differences between the performance achieved with a virtual prosthesis with respect to that obtained with a physical prosthesis were also evaluated. It is shown that the TAC test completion time correlated significantly with all physical outcome measures except the CRT. These results support the importance of reporting also the online performance metrics rather than only the offline classification error analysis [43,74].

The following studies [44,45,46,47] also reported a comparison of real-time performance between DC and PR based control strategies for SD and TH amputees: [44,45,46] only considered a physical prosthesis, while [47] presented a virtual prosthesis for controlling up to 2 DoFs. In most cases, the patients preferred either the more intuitive sequential or simultaneous pattern recognition control to the DC control, because this latter appeared to be unnatural and especially cumbersome for two DoFs tasks.

It is worth noticing that, with respect to the traditional PR control strategies, which were limited to sequentially controlling one DoF at the time, [43,45,47,59,71] also introduced the simultaneous PR control strategy, considered more promising because it showed improvements in throughputs and path efficiencies when compared to direct control or sequential PR. Moreover, the simultaneous PR control allowed for amputees to perform tasks with more than two DoFs with the lowest average completion times, completion rates and length error compared to the other control strategies, even if [43] argues that the simultaneous PR system has slightly lower performance with respect to the sequential PR system, but performs better than conventional DC control.

### Clinical Applications

In this section, the principal clinical outcomes of this review are discussed by focusing on the most relevant control strategies that allow a natural and simultaneous control of the prosthetic arm DoFs for TMR patients with different upper-limb amputations (i.e., BSD, SD, TH, and TR).

Firstly, for both the DC and PR strategies, the benefits of TMR surgery on the resulting control of different joints (as the elbow, wrist, and hand) have been introduced. As reported in almost all the analyzed papers [24,31,33,34,42,46,53], the patients can achieve better performance compared to the pre-TMR situation; furthermore, they also increase the ability to perform some ADLs, improving their quality of life.

These results confirmed that TMR provides new target muscle sites that are physiologically linked to the effective movements of the prosthetic device. However, in some studies [21,31,41], not all of the reinnervated sites are used as control sites due to the overlapping of EMG signals. In detail, the number of reinnervated sites is closely related to the number of DoFs that can be simultaneously controlled when using DC, whereas PR methods do not require mode-switching and independent EMG signals, even if almost all of the proposed classifiers only provide sequential control of multiple DoFs [13].

In order to examine the clinical robustness of myoelectric prosthetic control with TMR, the reported real-time performance metrics were taken into account to test whether the reinnervated muscles, after TMR surgery, can improve the myoelectric signals for real-time control of multifunction prostheses. Because of the lack of standard criteria, this review suggested a unified protocol test for the validation of these control strategies, by defining which tests are most suitable for the evaluation of prosthetic control for TMR patients with different amputation level.

Only the ACMC can be considered a standardized test for evaluating myoelectric control, because it has an established test-retest, inter-rater, and intra-rater reliability and clinical interpretation guidelines, as claimed by [68]. Additionally, as reported in [42], the following tests were also considered to be promising for assessing the functionality of a prosthesis: BBT, SHAP test, CRT, and JTHFT. Indeed, it can be noted that, in the analyzed works, the evaluation methods most commonly used are: BBT (modified up to 2 min) used in nine papers for a total of 34 subjects and CRT used in eight papers for a total of 33 subjects. Both of these tests have several advantages: they evaluate patient performance without differences among amputation levels or the number of reinnervated sites. Moreover, both tests are applicable for both DC and PR control, so that they were also used in the studies in which the two types of myoelectric control are compared. Furthermore, they can also be used on non-TMR patients to evaluate the performance of the prosthetic control, allow comparing the performance in pre and post-surgery situation, as in [21,24,31,33,34], to evaluate pre-and post-trial performance, as in [42], or to evaluate performance when different control strategies were used [44,46]. Lastly, these tests were used to compare performance in the control of prostheses with three or six DoFs [32]. Among the disadvantages, there is the fact that the above tests cannot be used in the absence of a prosthetic arm, therefore they cannot be used to evaluate the patient’s performance while using virtual reality. In [42], it is demonstrated that the TAC test completion time is a measure that significantly correlates with all the physical outcome measures, except the CRT.

For these reasons, we recommend, if possible, the use of Box and Block and Clothespin Relocation tests to evaluate the performance of the functional effectiveness of the prosthesis (with both DC and PR control) as a whole. In addition, this literature analysis also highlighted the need to have more quantitative information and to use instrumental indicators to be associated with these tests. For instance, when virtual reality is used with PR, real-time performance metrics that include motion selection time, motion completion time, and motion completion (“success”) rate can be considered for evaluating virtual arm movements.

Finally, regarding some clinical trials, we have also reported that the initial use of TMR was to prevent or treat phantom limb pain (PLP) and neuroma pain [25,27]. Other clinical investigations have focused on the role of the osseointegration [28] and the Targeted Sensory Reinnervation (TSR) [29] on TMR patients. Regarding the use of the osseointegration combined with TMR surgery, a direct interface that links the implant to the bone can provide even more stability when an external prosthesis is worn by amputees [19]. The TSR, instead, allows for controlling, in a bidirectional way, neuroprosthetic devices, thanks to the presence of a region with “sensory perception”.

## 8. Conclusions

This paper has provided an overview of the main advancements of the state of the art regarding prosthetic control techniques of the upper limb and performance evaluation methods for patients who have undergone TMR surgery.

Twenty papers were analyzed, highlighting that the most commonly used prosthetic control techniques are: in the context of direct Control, the proportional strategy and the method of simultaneous joint selection with co-contraction; in the context of PR methods, the LDA algorithm with various feature selection sets.

The most common performance evaluation methods, both for DC and PR, are BBT and CRT. In the case of prostheses controlled with PR, there is always the offline analysis of accuracy.

The most commonly used myoelectric prostheses are composed of 3 DoFs, for elbow F/E, wrist P/S, and hand O/C.

This work further highlighted the presence of a variety of tests that were used for the functional performance evaluation (Table 2), but there is a lack of standard criteria allowing to define which tests are the most suitable for the evaluation of prosthetic control for TMR patients with a different amputation level. In order to fill this gap, both the Box and Blocks and the Clothespin Relocation seem to be the most promising tests for evaluating the performance of the prosthetic systems.

In addition, we believe that virtual reality can be used to further explore the potentiality of the proposed control approaches, before considering them on a physical prosthesis. In fact, we proposed extending the use of virtual reality performance indexes, defined for PR, like motion selection time, motion completion time, and motion completion (“success”) rate also to the DC control in this way: the motion selection time and motion completion time can be modified by considering the time that is required from EMG onset to remain above the threshold, while the success rate does not need to be modified. In this way, a comparative analysis between DC and PR systems can also be done when using a virtual reality system.

It has to be noted that only few articles presented results regarding the simultaneous PR control strategy showing improvements in throughputs and path efficiencies when compared to direct control or sequential PR [43,45,47,59,71]. Thus, the possibility of simultaneously controlling the prosthesis still can be improved with simultaneous PR-based controllers.

In conclusion, despite the great progresses in the field of advanced prosthetic control, this paper highlights the necessity to still identify the best PR/DC-based system allowing for robust control when considering more than 2 DoFs and of defining standard evaluation methods of the real-time control strategy performance.

## Figures and Tables

**Table 2:** Performance evaluation methods.

Test Used	Percentage of Enrolled Patients (%)	N° of Articles
Qualitative/subjective evaluation (questionnaire)	20	7
Box and Block	50	9
Clothespin Relocation	49	8
Wolf Motor Functions—WMFT	1	1
Assessment of Motor and Process Skills—AMPS	14	3
Cubbies	1	1
Cups	1	1
Target Achievement Control—TAC	19	2
Southampton Hand Assessment Procedure—SHAP	25	2
Accuracy offline	35	8
Classification Error rate	35	5
Assessment of Capacity for Myoelectric Control—ACMC	25	2
Real Time Virtual Test	14	3
Action Research Arm Test—ARAT	1	1
Jebsen-Taylor Test of Hand Function—JTHFT	13	1
Block stacking	5	1
Fitts’ Target Acquisition Task—FTAT	1	1

**Table 3:** Evaluation of the performance obtained with the DC and Pattern Recognition (PR) systems reported in the papers presented in Section 6.3.

Performance Evaluation Method	Metric Indicators	DC	PR		Study
**BBT**	Number of 1-inch blocks moved over a barrier in two min (average value on 4 TMR patients)	10.7 ± 4.3	15 ± 3		[44]
	Number of 1-inch blocks moved over a barrier in two min (average value on 8 TMR patients)	15.6 ± 2.7	13.4 ± 2.6		[46]
**CRT**	Time (s) to move three clothespins (average value on 4 TMR patients)	60 ± 15	45 ± 11		[44]
	Time (s) to move three clothespins (average value on 8 TMR patients)	137 ± 60.2	90.2 ± 39.6		[46]
**ACMC**	Test score (average value on 8 TMR patients)	44.4 ± 3.4	47.3 ± 3.9		[46]
**SHAP**	Index of function (average value on 8 TMR patients)	18 ± 5	31 ± 3		[46]
**TAC**	Completion Time (s) 1 DoF (average value on 4 TMR patients)	2.651.66	1.4 ± 0.3 (Seq)	2.03 ± 0.83 (Sim)	[47]
	Completion Time (s) 2 DoF (average value on 4 TMR patients)	3.55 ± 1.66	3.6 ± 0.42 (Seq)	1.93 ± 0.82 (Sim)	
	Completion rate (%) 1 DoF (average value on 4 TMR patients	86.25	100 (Seq)	93.75 (Sim)	
	Completion rate (%) 2 DoF (average value on 4 TMR patients)	81.25	92.5 (Seq)	98.75 (Sim)	
	Length error (%) 1 DoF (average value on 4 TMR patients	97.4 ± 81	13.96 ± 4.65 (Seq)	32 ± 29.52 (Sim)	
	Length error (%) 2 DoF (average value on 4 TMR patients)	86.65 ± 52.77	67.88 ± 11.8 (Seq)	21.88 ± 25.08 (Sim)	
**FTAT**	Throughput (bit/s) (1 DoF)	2.64 ± 0.24	3.67 ± 0.23 (Seq)	2.11 ± 0.18 (Sim)	[45]
	Throughput (bit/s) (2 DoF)	1.24 ± 0.04	1.32 ± 0.03 (Seq)	1.63 ± 0.05 (Sim)	
	Path efficiency (%) (1 DoF)	90.1 ± 0.23	97.00 ± 0.96 (Seq)	96.3 ± 1.12 (Sim)	
	Path efficiency (%) (2 DoF)	71.3 ± 0.80	71.60 ± 0.76 (Seq)	87.7 ± 0.7 (Sim)	

## Abbreviations

The following abbreviations, reported in alphabetic order, are used in this manuscript:

ACMC	Assessment for Capacity of Myoelectric Control
ADL	Activities of Daily Living
AMPS	Assessment of Motor and Process Skills
AR	Auto Regressive
ARAT	Action Research Arm Test
BBT	Box and Block Test
BSD	Bilateral Shoulder Disarticulation
CRT	Clothespin Relocation Test
DC	Direct Control
DoF	Degree of Freedom
EMG	ElectroMyoGraphy
ESN	Echo State Network
F/E	Flexion/Extension
FSR	Force Sensor Resistor
HD	High Density
HT	Higher Threshold
I/E	Intra/Extra
imEMG	intramuscolarEMG
JTHFT	Jebsen-Taylor test of Hand Function
LDA	Linear Discriminant Analysis
MAV	Mean Absolute Value
mcr	motion completion rate
mct	motion completion time
MCU	MicroController Unit
MLP	Multi Layer Perceptron
mst	motion selection time
NLR	Nonlinear Logistic Regression
O/C	Open/Close
P/S	Pronation/Supination
PLP	Phantom Limb Pain
PR	Pattern Recognition
PRISMA	Preferred Reporting Items for Systematics reviews and Meta-Analyses
RMS	Root Mean Square
ROM	Range Of Motion
SD	Shoulder Disarticulation
sEMG	surfaceEMG
seqPR	sequential PR
SHAP	Southampton Hand Assessment Procedure
simPR	simultaneous PR
SSC	Slope Sign Changes
ST	Standard Threshold
SVM	Support Vector Machine
TAC	Target Achievement Control
TD	Time Domain
TD-AR	Time Domain and Auto Regressive
TH	Trans-Humeral
TMR	Targeted Muscle Reinnervation
TSR	Targeted Sensory Reinnervation
VR	Virtual Reality
WL	Waveform Length
WMFT	Wolf Motor Functions Tests
ZC	Zero Crossing
